# Cancer-testis antigen CEP55 serves as a prognostic biomarker and is correlated with immune infiltration and immunotherapy efficacy in pan-cancer

**DOI:** 10.3389/fmolb.2023.1198557

**Published:** 2023-07-07

**Authors:** Xiaodong Xie, Hongyin Liang, Wushuang Jiangting, Yu Wang, Xiao Ma, Zhen Tan, Long Cheng, Zhulin Luo, Tao Wang

**Affiliations:** ^1^ Department of General Surgery, The General Hospital of Western Theater Command, Chengdu, Sichuan, China; ^2^ Department of General Surgery and Pancreatic Injury and Repair Key Laboratory of Sichuan Province, The General Hospital of Western Theater Command, Chengdu, Sichuan, China; ^3^ Department of Anesthesiology, The General Hospital of Western Theater Command, Chengdu, Sichuan, China; ^4^ Department of Microbiology, Zunyi Medical University, Zunyi, Guizhou, China; ^5^ College of Medicine, The Southwest Jiaotong University, Chengdu, Sichuan, China

**Keywords:** CEP55, pan-cancer, prognostic biomarker, immunotherapy efficiency, CMAP, molecular docking

## Abstract

**Background:** Centrosomal Protein 55 (CEP55) was initially described as a main participant in the final stage of cytokinesis. Further research identified CEP55 as a cancer-testis antigen (CTA) that is aberrantly expressed in different malignancies and a cancer vaccination candidate. The current study aimed to disclose the complete expression of CEP55, its effect on various malignancy prognoses, and its role in the tumor microenvironment.

**Methods:** Transcriptional information regarding tumor and normal tissues, as well as externally validated and protein expression data were gathered from the Cancer Genome Atlas, Genotype-Tissue Expression project, Gene Expression Omnibus, and Human Protein Atlas. We examined the effect of CEP55 on tumor prognosis using Kaplan-Meier (KM) and univariate Cox regression analyses. In addition, we investigated the connections between CEP55 expression and hallmark cancer pathways, immune cell infiltration, and immune regulator expression across malignancies. We constructed and validated a CEP55-related risk model for hepatocellular carcinoma (HCC) and explored the correlations between CEP55 expression and HCC molecular subtypes. Finally, we investigated putative small-molecule drugs targeting CEP55 using a connectivity map (CMap) database and validated them using molecular docking analysis.

**Findings:** CEP55 was aberrantly expressed in most cancers and revealed a prognostic value for several malignancies. Cancers with high CEP55 expression showed significantly enhanced cell cycle, proliferation, and immune-related pathways. For most malignancies, elevated CEP55 expression was associated with the infiltration of myeloid-derived suppressor cells (MDSCs) and Th2 cells. In addition, CEP55 expression was linked to immunomodulators and the potential prediction of immune checkpoint inhibitor (ICI) responses, and strongly associated with distinct molecular HCC subtypes, whereby the CEP55-based nomogram performed well in predicting short- and long-term HCC survival. Finally, we used connectivity map (CMap) and molecular docking analyses to discover three candidate small-molecule drugs that could directly bind to CEP55.

**Conclusion:** CEP55 affected the occurrence and development of various cancers and possibly the regulation of the tumor immune microenvironment. Our findings suggest that CEP55 is a potential biomarker for prognosis and a powerful biomarker for ICI efficacy prediction.

## 1 Introduction

The considerable increase in cancer incidence and mortality in recent decades has caused this disease to become the primary cause of death and a main factor in the decrease in life expectancies worldwide (Sung et al., 2021). Approximately 1,958,310 million new cancer cases and 609,820 cancer-related deaths are expected in the United States in 2023 (Siegel et al., 2023). Although targeted therapy and immunotherapy advances have greatly improved tumor treatment, the long-term survival rates of patients with various forms of tumors are unsatisfactory (Hughes et al., 2016; Sharma et al., 2017). Novel, sensitive diagnostic markers and suitable therapeutic targets are critical for improving prognoses for cancer patients.

Various proteins are involved in the pathogenesis and progression of cancers, including survival-associated signaling kinases with relatively common mutants (e.g., BRAF V600E and KRAS G12D) (Cisowski et al., 2016), DNA damage repair response molecules (e.g., p53 and Rad51) (Bonilla et al., 2020), and cell cycle progression-associated compounds (e.g., Rb1 and CDK4/6) (Fassl et al., 2022). In particular, the targeting of cell cycle progression has advanced greatly with the development of CDK4/6 inhibitors that are now in clinical use for HER2+ breast cancer and are under investigation for the treatment of a variety of Rb1+ cancers (Xu et al., 2017; Pesch et al., 2022). However, these treatments are ineffective for the large proportion of cancers that have CDK4/6 or Rb1 mutations. Therefore, cell cycle inhibitors must be sourced that can be used in these contexts.

Centrosomal Protein 55 (CEP55, also known as c10orf3 and FLJ10540) was initially described in physiological studies as a midbody- and centrosome-associated coiled-coil protein with a size of approximately 55 kDa (Fabbro et al., 2005). Aberrant expression of CEP55 has been strongly associated with clinical features and prognoses in cancer patients, which has led to an increasing interest in this molecule as a possible therapeutic target (Jeffery et al., 2016). In recent years, this interest has led to the identification of multiple independent molecular mechanisms that explain CEP55 activity. CEP55 interacts with endosomal sorting components to recruit the endosomal sorting complex required for transport (ESCRT) to the midbody, and may play a significant role in the constriction of the intracellular bridge and the promotion of abscission and cytokinesis (Zhao et al., 2006). Defects in cytokinesis can cause the generation of aneuploid cells, which is a key step in tumorigenesis; therefore, CEP55 is suggested to be involved in tumor initiation (Lens and Medema, 2019). In addition, CEP55 binds and stabilizes the PI3K catalytic subunit and facilitates the activation of the PI3K/Akt pathway, thus promoting the survival and proliferation of cancer cells (Montero-Conde et al., 2008). This association offers an alternative mechanism for CEP55 to participate in the later stages of tumor progression and metastasis. Beyond these cell division and signaling roles, CEP55 has recently been identified as an immunogenic tumor-associated antigen (TAA) and cancer-testis antigen (CTA), which recommend it as a potential candidate for cancer vaccine therapies. CEP55 peptides are naturally present in breast cancer cells and can induce an increase in antigen-specific cytotoxic T lymphocytes (CTLs) (Inoda et al., 2009). CEP55-specific CTLs could be capable of recognizing and killing chemotherapy-resistant colon cancer stem cells (Inoda et al., 2011; Gao and Wang, 2015). A recent study revealed that CEP55 is expressed in exosomes derived from malignant cells and has the potential to be a non-invasive diagnostic marker for tumors (Qadir et al., 2018). Collectively, the results of these studies demonstrate that CEP55 could have various functions in multiple cancers. However, a comprehensive picture of CEP55 in the tumor immune microenvironment has not yet been reported, and small therapeutic compounds targeting CEP55 remain elusive.

Through an integrated analysis of the genomic and expression data of multiple independent databases, this study aimed to thoroughly characterize the functions of CEP55 in the regulation of tumor biological processes and the immune microenvironment and its potential as an immunotherapy and chemotherapy target at the pan-cancer level. CEP55 was aberrantly expressed in most cancers and showed prognostic value for several malignancies. High CEP55 expression was strongly correlated with the infiltration of myeloid-derived suppressor cells (MDSCs) and Th2 cells in most cancers, and was substantially related to distinct molecular subtypes of HCC, whereby the CEP55-based nomogram performed well in predicting short- and long-term HCC survival. Finally, using a connectivity map (CMap) and molecular docking analyses, we identified three potential small molecules targeting CEP55, which may mitigate the immunosuppressive microenvironment and enhance the anti-tumor effect of ICIs.

## 2 Materials and methods

### 2.1 Data acquisition and processing

Gene expression data and pathological and clinical information from The Cancer Genome Atlas (TCGA) project and normal samples from the Genotype-Tissue Expression (GTEx) dataset were downloaded from UCSC Xena (https://xenabrowser.net/). This study comprehensively analyzed 33 types of cancer from 9,807 tumor tissues from TCGA database and 7,873 normal tissues (727 from TCGA database and 7,146 from the GTEx database). Batch effects were corrected using the UCSC TOIL RNA-seq Recompute workflow as previously described (Vivian et al., 2017). Transcripts per kilobase million (TPM) normalized expression data were used for subsequent analyses. Microarray expression data of 29 cancers were downloaded from the GEO database (http://www.ncbi.nih.gov/geo/) for external validation. HCC gene expression data from the ICGC (https://dcc.icgc.org/) database (including 197 normal tissues and 240 HCC tumor tissues) and the GSE14520 dataset (including 239 normal tissues and 247 HCC tumor tissues) were included in the validation of CEP55 in HCC. Information on the datasets used in this study is detailed in Supplementary Table S1.

### 2.2 CEP55 protein expression, localization, and interaction

The Human Protein Atlas (HPA; www.proteinatlas.org) initiative uses multiple omics methods to focus on protein expression in cells, tissues, and organs (Uhlén et al., 2015). The expressions of CEP55 in normal tissues and cell types were obtained using the “Tissue” and “Single Cell Type” sections of this atlas. The “Pathology” section was used to validate the protein levels of CEP55 (HPA023430) in various cancers. Immunofluorescence staining images from the “Subcellular” section were used to explore the subcellular localization of CEP55. The protein-protein interactions of CEP55 were analyzed using the ComPPI website (https://comppi.linkgroup.hu/), which provides integrated information regarding protein-protein interactions and their localization (Veres et al., 2015).

### 2.3 Genomic alteration analysis of CEP55

The cBioPortal site (http://www.cbioportal.org/) is a public open-access link to multidimensional cancer genomic features, including genetics, epigenetics, gene expression, and proteomics (Cerami et al., 2012; Gao et al., 2013). The CEP55 genome alterations and their relationships with survival were determined using the “Mutation,” “Plots” and “Cancer Types Summary” modules of this portal. Since microsatellite instability and tumor mutation burden (MSI and TMB, respectively) can predict responses to immunotherapy, MSI scores were obtained from the somatic mutation data. TMB scores were calculated using a Perl script and modified by dividing the scores by the total length of the exons. Spearman’s correlation coefficient was used to assess the relationship between CEP55 expression and TMB or MSI.

### 2.4 Tumor immune infiltration analysis

The “Estimate” method infers the fraction of infiltrating stromal and immune cells in tumor samples (Yoshihara et al., 2013). The R package “estimate” was utilized to compute the “Immune Score,” “Stromal Score,” “Tumor Purity” and “Estimate Score” in this study. The Tumor Immune Estimation Resource (TIMER) database provides a comprehensive resource for analyzing the abundances of infiltrated immune cells across cancers using various immune deconvolution methods (Li et al., 2017; Li et al., 2020). The correlations between CEP55 expression and pan-cancer immune infiltration levels were accessed from the “Immune Association” section of the TIMER2.0 database (http://timer.cistrome.org/).

### 2.5 Functional and pathway enrichment analysis

The patients with CEP55 mRNA expression levels in the highest 30% were referred to as the high-CEP55 group and those with the lowest 30% were referred to as the low-CEP55 group. The “limma” R package was used to determine the different expression genes (DEGs) of the high- and low-CEP55 subgroups (Ritchie et al., 2015). The cancer HALLMARK geneset (h.all.v7.2. symbols) was used for the functional and pathway enrichment analyses. The normalized enrichment score (NES) and false discovery rate (FDR) for each cancer type were determined using the R package “clusterProfiler” (Yu et al., 2012).

### 2.6 Immunotherapy prediction analysis

Immunomodulators, which contain various immunoregulatory genes, are critical components of immunotherapy treatments (Thorsson et al., 2018). Spearman’s correlation coefficients between CEP55 expression and the immunomodulators were calculated for each cancer type. To explore the influence of CEP55 in the response to ICIs, CEP55 expression differences between responders and non-responders were calculated for nine independent immunotherapy cohorts. Detailed information on each immunotherapy cohort is provided in Supplementary Table S2.

### 2.7 Connectivity map for specific inhibitor analysis

The Connectivity Map (Cmap; https://clue.io/) database contains millions of gene expression profiles from different cell types that have been treated with perturbagens and is commonly used to predict cellular responses to chemical stimuli (Lamb et al., 2006). The 150 main up- or downregulated DEGs for each cancer type were subjected to Cmap analysis. Detailed lists of the query results were acquired and the scores were used to perform a heatmap analysis.

### 2.8 Molecular docking analysis

The 3D structure of CEP55 was downloaded from the PDB Data Bank (http://www.rcsb.org/), and the structural formulae of the small compounds were obtained from PubChem (https://pubchem.ncbi.nlm.nih.gov/). AutoDockTools (v1.5.7) software was used for the molecular docking analysis. A docking free energy of ≤ −7.0 kcal/mol was considered stable binding. The molecular docking results were visualized using PyMOL software (v2.4.0) and the Protein Plus online server (https://proteins.plus/) (Schöning-Stierand et al., 2020).

### 2.9 Survival analysis

Overall survival (OS) and disease-specific survival (DSS) were used to determine the prognostic value of CEP55 expression across the various cancer types. Patients were separated into low- and high-CEP55 expression groups using the median CEP55 expression as the cutoff value. The “survival” and “forestplot” R packages were used to conduct a Cox regression analysis for pan-cancer. The Kaplan-Meier method and log-rank test were used for the survival analysis.

### 2.10 Statistical analyses

All computational procedures and statistical analyses were conducted using R software (https://www.r-project.org). Two normally distributed groups were analyzed for statistical significance using the unpaired Student’s *t*-test, and non-normally distributed groups were compared using the Wilcoxon rank-sum test. The chi-squared or Fisher’s exact test was used to compare the contingency table variables, and the log-rank test was used to compare the results of the survival analysis using Kaplan-Meier method. Statistical significance was defined as *p* < 0.05.

## 3 Results

### 3.1 The expression and localization of CEP55 in normal tissues and cells

**FIGURE 1:**
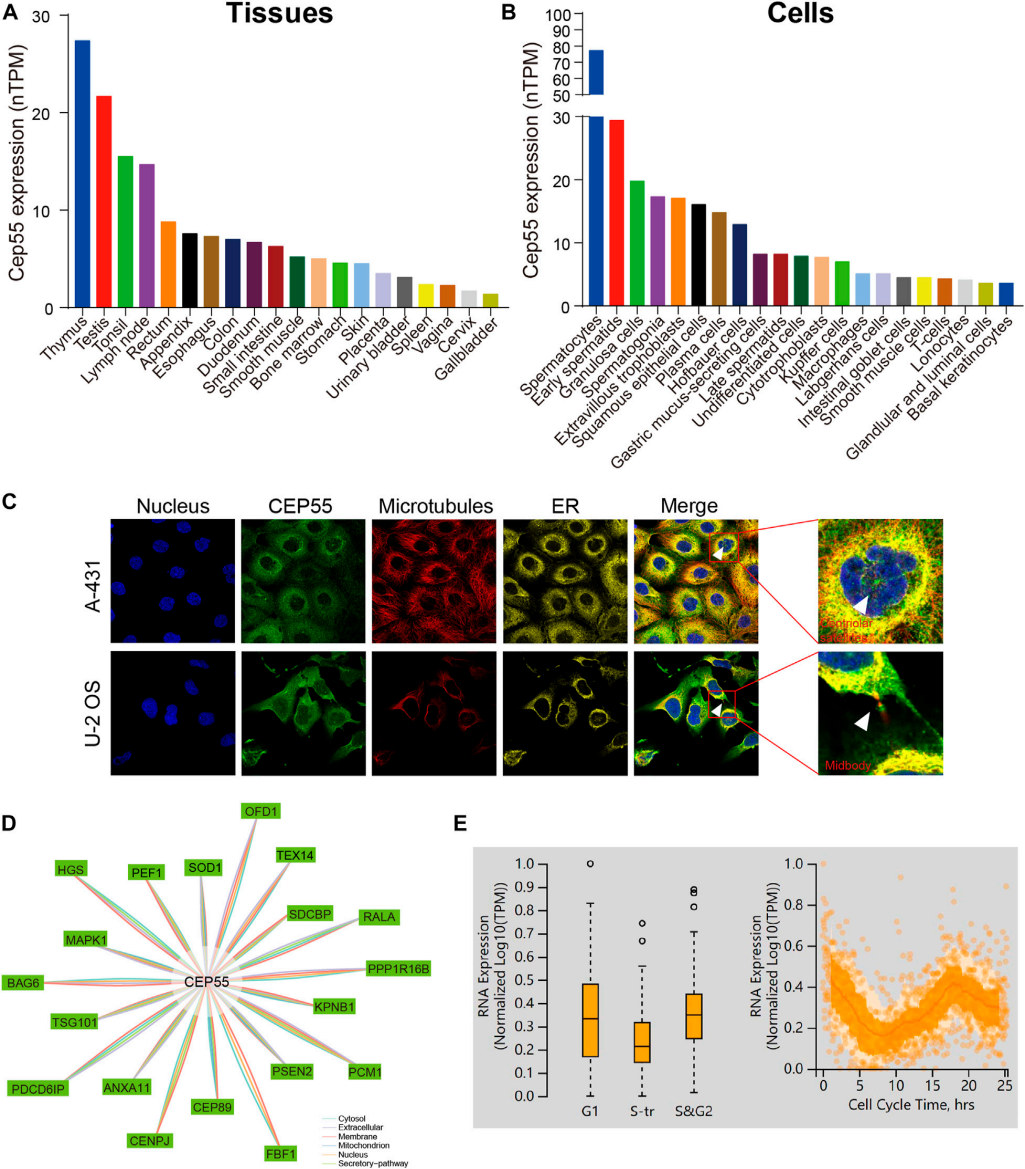
CEP55 expression and localization in normal tissues and cells. **(A)** The expression of CEP55 in normal tissues. **(B)** The expression of CEP55 in normal cells. **(C)** Subcellular localization of CEP55 in A-431 and U251 cell lines. The images were retrieved from the HPA (www.proteinatlas.org) database. **(D)** Protein-protein interaction (PPI) network of CEP55. **(E)** The correlation between CEP55 mRNA expression and cell cycle progression. The picture was obtained from the HPA (www.proteinatlas.org) database.

We first analyzed CEP55 expression in tissues and cells using the HPA database and enhanced expression was observed in the testis and lymphoid tissues such as the thymus, tonsils, and lymph nodes. At the cellular level, CEP55 was highly expressed in germ cells (spermatocytes, early spermatids, and spermatogonia), epithelial cells (granulosa cells and squamous epithelial cells), and immune-related cells (plasma cells and Hofbauer cells) (Figures 1A,B). The immunofluorescence (IF) images of the HPA database showed that CEP55 was mainly located in the plasma membrane, centriolar satellites, and midbody region (Figure 1C). CEP55 interacted proteins were localized in the cytosol, extracellular space, membrane, mitochondria, nucleus, and secretory pathway (Figure 1D). Based on the fluorescent ubiquitination-based cell cycle indicator (FUCCI) assay and single-cell RNA sequences from the HPA database, CEP55 showed a strong correlation with the cell cycle and variable peak expression across cell cycle phases, which are consistent with its function in cytokinesis (Figure 1E).

### 3.2 Aberrant expression of CEP55 in cancer tissues

**FIGURE 2:**
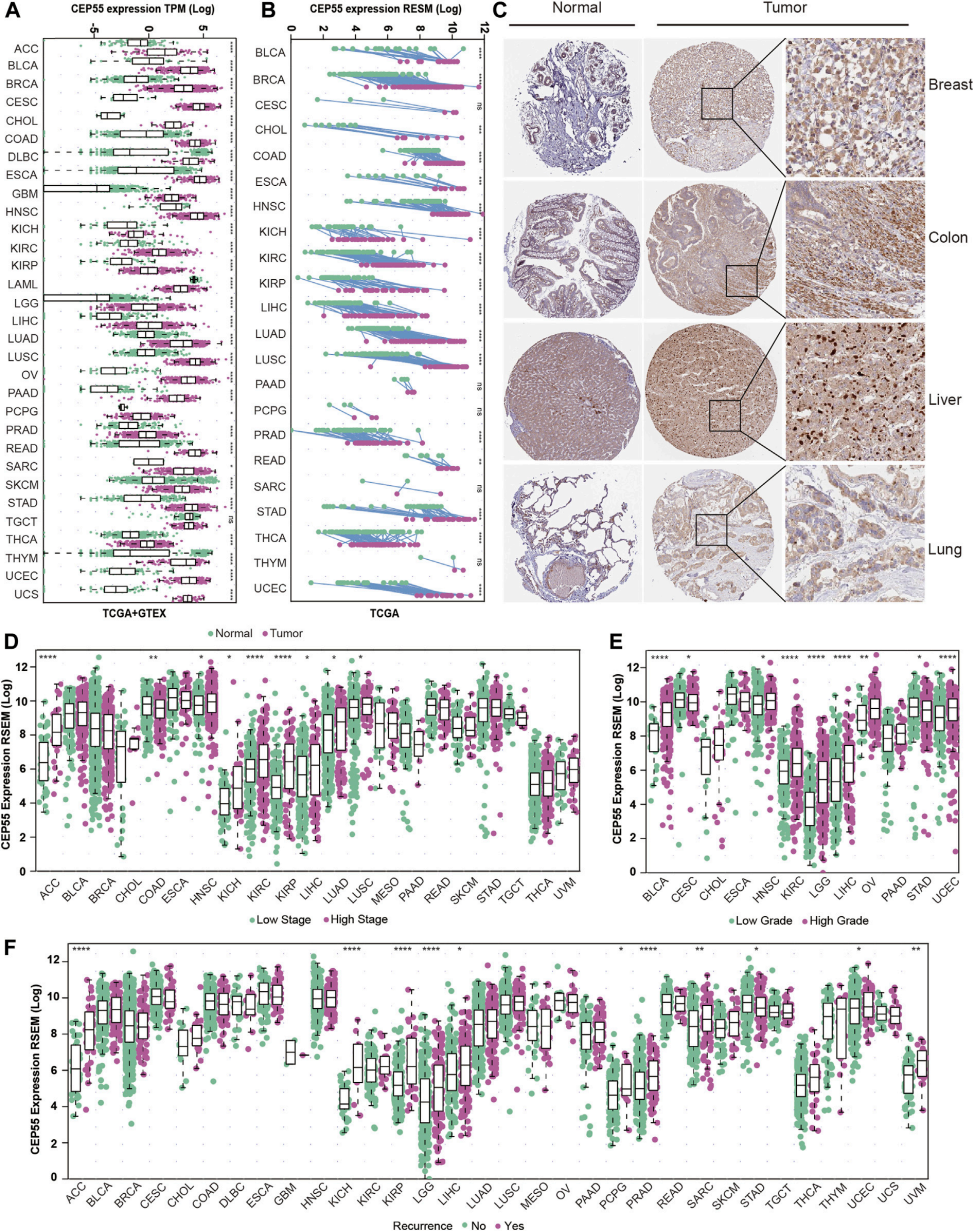
CEP55 expression in human cancers. **(A)** CEP55 expression in tumor and normal tissues based on the integrated data from TCGA and GTEx datasets. **(B)** The expression of CEP55 in tumor and paired tumor-adjacent normal tissues based on the TCGA dataset. **(C)** Immunohistochemical staining of CEP55 in cancers. The images are from the HPA (www.proteinatlas.org) database. **(D)** The expression of CEP55 in high- and low-stage tumors. **(E)** The expression of CEP55 in high- and low-grade tumors. **(F)** The expression of CEP55 in recurrent and non-recurrent tumors. Asterisks indicate statistical *p*-values (ns, *p* > 0.05, *p < 0.05, **p < 0.01, ***p < 0.001, and ****p < 0.0001).

To further investigate the critical role of CEP55, we analyzed its expression in various cancers. Through integrated GTEx-TCGA data analysis, CEP55 showed significantly higher expression in tumor tissues than normal tissues in approximately 30 cancers, 23 of which were verified by external datasets: adrenocortical cancer (ACC), breast invasive carcinoma (BRCA), bladder urothelial carcinoma (BLCA), colon adenocarcinoma (COAD), cervical and endocervical cancer (CESC), cholangiocarcinoma (CHOL), diffuse large B-cell lymphoma (DLBC), glioblastoma multiforme (GBM), esophageal carcinoma (ESCA), kidney chromophobe (KICH), kidney papillary cell carcinoma (KIRP), kidney clear cell carcinoma (KIRC), liver hepatocellular carcinoma (LIHC), lung squamous cell carcinoma (LUSC), lung adenocarcinoma (LUAD), ovarian serous cystadenocarcinoma (OV), rectum adenocarcinoma (READ), pancreatic adenocarcinoma (PAAD), skin cutaneous melanoma (SKCM), sarcoma (SARC), stomach adenocarcinoma (STAD), thyroid carcinoma (THCA), and uterine corpus endometrioid carcinoma (UCEC) (Figure 2A; Supplementary Figure S1). Furthermore, CEP55 expression was considerably higher in cancer tissues than in the corresponding tumor-adjacent normal tissues in 17 cancers: BRCA, BLCA, CHOL, ESCA, COAD, HNSC, KICH, KIRP, KIRC, LIHC, LUSC, LUAD, prostate adenocarcinoma (PRAD), STAD, READ, THCA, and UCEC (Figure 2B). At the protein level, immunohistochemistry (IHC) data from the HPA database revealed that CEP55 expression was considerably greater in cancer tissues than in normal tissues (Figure 2C). Therefore, we assessed the correlation between CEP55 expression and clinical characteristics and found that CEP55 expression was greater in high-stage tumors (Stages III and IV) than in low-stage tumors (Stages I and II) of ACC, HNSC, KICH, KIRP, KIRC, LIHC, LUAD, and LUSC, whereas CEP55 expression was lower in high-stage than low-stage COAD tumors (Figure 2D; Supplementary Figure S2A). CEP55 was elevated in high-grade (Grades III and IV) malignancies in BLCA, KIRC, HNSC, LIHC, LGG, OV, and UCEC, but showed reduced expression in high-grade CESC and STAD tumors (Figure 2E; Supplementary Figure S2B). Moreover, patients with recurrences during follow-up showed increased expression of CEP55 in ACC, KIRP, KICH, LIHC, LGG, PRAD, pheochromocytoma and paraganglioma (PCPG), UCEC, SARC, and uveal melanoma (UVM), and reduced expression in STAD (Figure 2F). These findings indicate that CEP55 is frequently overexpressed in multiple cancers and is associated with clinical characteristics.

### 3.3 CEP55 correlates with prognosis in pan-cancer

**FIGURE 3:**
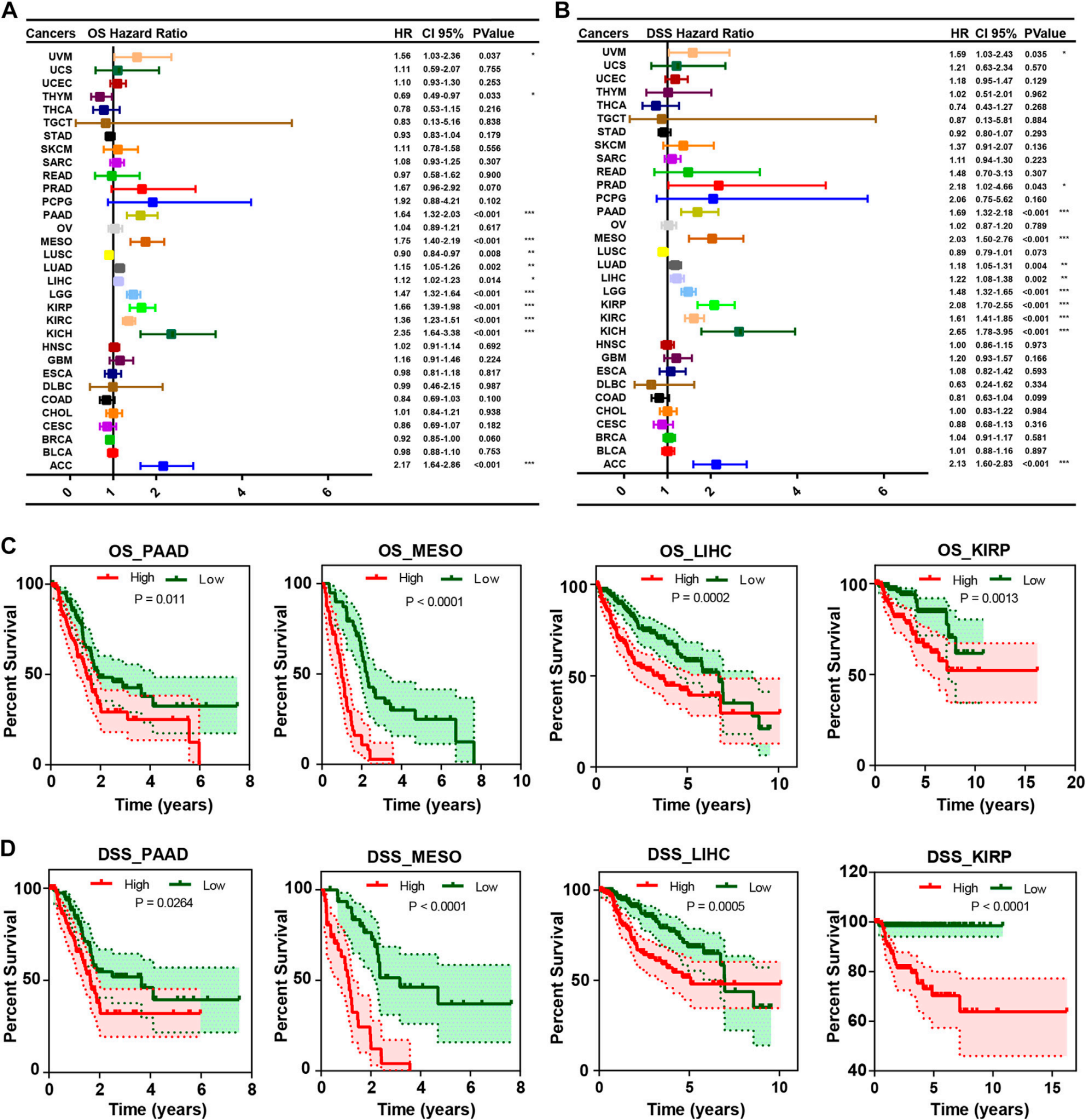
The correlation between CEP55 expression and overall survival (OS) and disease-specific survival (DSS). **(A)** The correlation between CEP55 expression and overall survival (OS). **(B)** The correlation between CEP55 expression and disease-specific survival (DSS). **(C)** Kaplan-Meier overall survival curves of CEP55 in PAAD, MESO, LIHC, and KIRP. **(D)** Kaplan-Meier disease-specific survival curves of CEP55 in PAAD, MESO, LIHC, and KIRP. Asterisks indicate statistical *p*-values (ns, *p* > 0.05, *p < 0.05, **p < 0.01, and ***p < 0.001).

To assess the relationship between CEP55 expression and pan-cancer prognosis, we estimated overall survival (OS) and disease-specific survival (DSS) using univariate Cox regression with TCGA dataset. Increased expression of CEP55 showed significant correlations with poor OS rates in UVM (HR = 1.56, *p* = 0.037), PAAD (HR = 1.64, *p* < 0.001), MESO (HR = 1.75, *p* < 0.001), LUAD (HR = 1.15, *p* = 0.002), LIHC (HR = 1.12, *p* = 0.014), LGG (HR = 1.47, *p* < 0.001), KIRP (HR = 1.66, *p* < 0.001), KIRC (HR = 1.36, *p* < 0.001), KICH (HR = 2.35, *p* < 0.001), and ACC (HR = 2.17, *p* < 0.001). Higher CEP55 expressions predicted better OS rates in LUSC (HR = 0.90, *p* = 0.008) and THYM (HR = 0.69, *p* = 0.033) (Figure 3A). Higher expression of CEP55 was also related to poor DSS in UVM (HR = 1.59, *p* = 0.035), PRAD (HR = 2.18, *p* < 0.043), PAAD (HR = 1.69, *p* < 0.001), MESO (HR = 2.03, *p* < 0.001), LUAD (HR = 1.18 *p* = 0.004), LIHC (HR = 1.22, *p* = 0.002), LGG (HR = 1.48, *p* < 0.001), KIRP (HR = 2.08, *p* < 0.001), KIRC (HR = 1.61, *p* < 0.001), KICH (HR = 2.66, *p* < 0.001), and ACC (HR = 2.13, *p* < 0.001) (Figure 3B; Supplementary Table S3). KM survival curves showed better OS in the low-CEP55 expression group for PAAD (HR = 1.71, *p* = 0.011), MESO (HR = 4.86, *p* < 0.001), LIHC (HR = 2.00, *p* = 0.001), KIRP (HR = 2.64, *p* = 0.0013), ACC (HR = 8.63, *p* < 0.001), LGG (HR = 3.07, *p* < 0.001), KIRC (HR = 1.91, *p* < 0.001), LUAD (HR = 1.51, *p* = 0.003), and PRAD (HR = 7.07, *p* = 0.031); however, high OS was observed in the high-CEP55 group for THYM (HR = 0.21, *p* = 0.030) and STAD (HR = 0.67, *p* = 0.009) (Figure 3C; Supplementary Figure S3A). In PAAD (HR = 1.70, *p* = 0.026), MESO (HR = 6.5, *p* < 0.001), LIHC (HR = 2.21, *p* < 0.001), KIRP (HR = 6.26, *p* < 0.001), ACC (HR = 8.01, *p* < 0.001), BRCA (HR = 1.54, *p* = 0.038), KIRC (HR = 2.78, *p* < 0.001), LGG (HR = 3.30, *p* < 0.001), LUAD (HR = 1.68, *p* = 0.003), PRAD (HR = 6.48, *p* = 0.038), and UVM (HR = 2.56, *p* = 0.034), the low-CEP55 expression group had higher DSS, while for STAD (HR = 0.59, *p* = 0.012), the high expression group showed better DSS rates (Figure 3D; Supplementary Figure S3B). These results indicate that higher CEP55 expression is generally associated with worse prognoses for the majority of tumors.

### 3.4 Mutation landscape of CEP55 in pan-cancer

**FIGURE 4:**
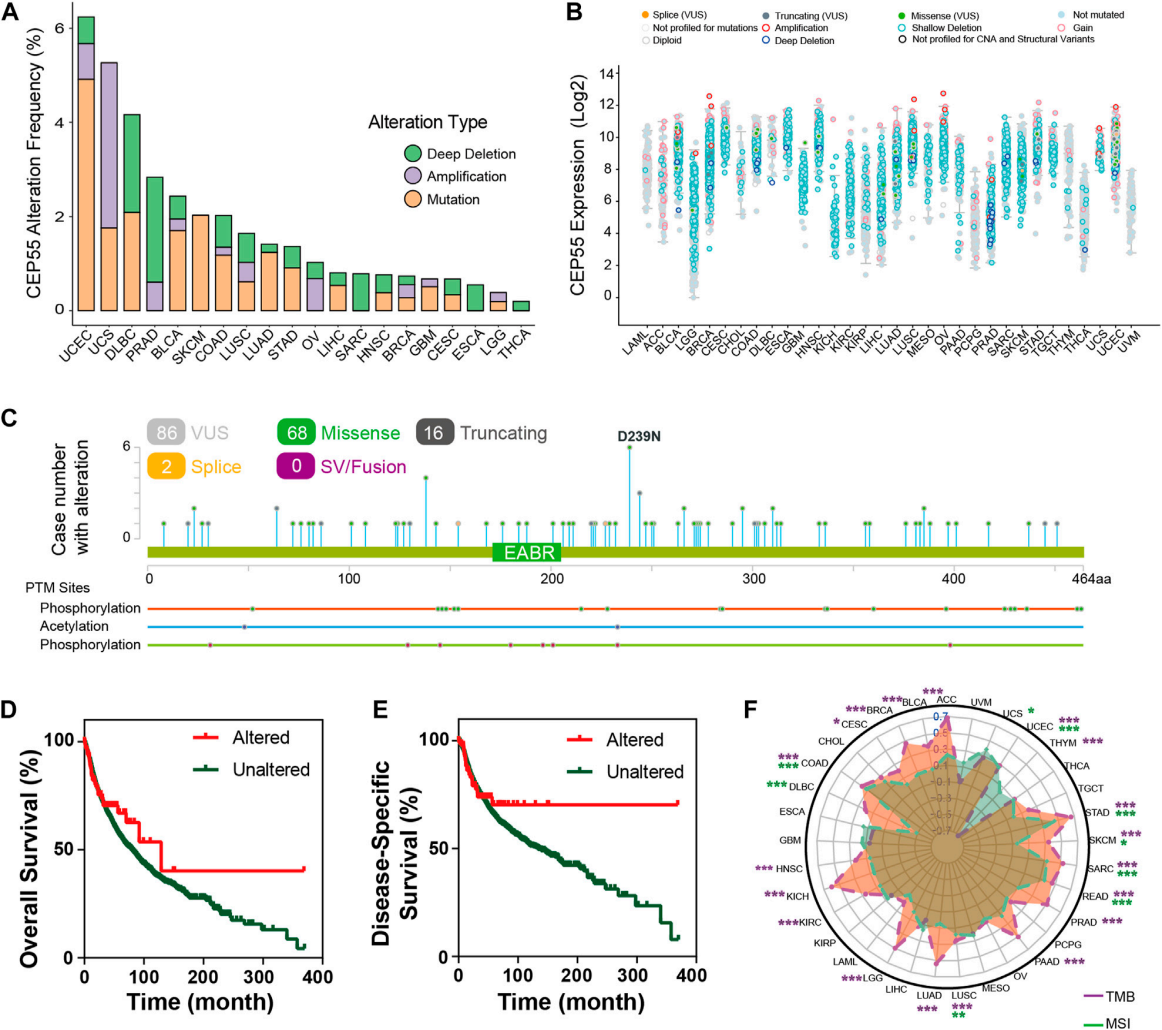
Mutation characteristics of CEP55 in cancers. **(A)** The alteration frequencies of CEP55 in various cancers. **(B)** The mutation counts of CEP55 in various cancers. **(C)** The mutation sites of CEP55. **(D)** Kaplan–Meier curve for OS in the altered and unaltered groups. **(E)** Kaplan–Meier curve for DSS in the altered and unaltered groups. **(F)** The correlations between CEP55 expression and TMB (purple) and MSI (green). Asterisks indicate statistical *p*-values (ns, *p* > 0.05, *p < 0.05, and **p < 0.01).

We inspected the mutation profiles of CEP55 using the cBioPortal database to investigate genomic alterations. Genetic variations in CEP55 were detected in 20 of the 33 cancer types. The highest alteration frequency was observed with UCEC, with 6.24% of all UCEC cases (Figure 4A). “Mutation” was the primary variation type in most of the cancers, such as UCEC, BLCA, and SKCM, while “amplification” and “deep deletion” were predominant in other cancers, such as UCS, PRAD, and SARC. The relationships between alteration type and CEP55 expression in pan-cancer are shown in Figure 4B. A total of 86 putative non-synonymous mutation sites were detected between amino acids 0 and 464, including 68 missenses, 16 truncating, and 2 splice variants (Figure 4C). Moreover, patients with CEP55 variation showed a trend toward better prognosis in terms of OS and disease-free survival (DFS) (Figures 4D,E). Tumor mutation burden (TMB) and microsatellite instability (MSI) are associated with the occurrence and development of malignancies and are regarded as predictive markers for responses to immunotherapy. We then analyzed the relationships between CEP55 expression and TMB/MSI in all cancers. CEP55 expression was positively correlated with TMB in UCEC, STAD, SARC, SKCM, READ, PAAD, PRAD, LUSC, LGG, LUAD, KICH, KIRC, COAD, HNSC, CESC, BLCA, BRCA, and ACC, and negatively correlated with TMB in THYM. In addition, the expression of CEP55 was positively associated with MSI in UCS, UCEC, STAD, COAD, SARC, READ, LUSC, and DLBC, but these were negatively correlated in SKCM and DLBC (Figure 4F, *p* < 0.05; Supplementary Table S4).

### 3.5 Functional analysis based on CEP55 expression

**FIGURE 5:**
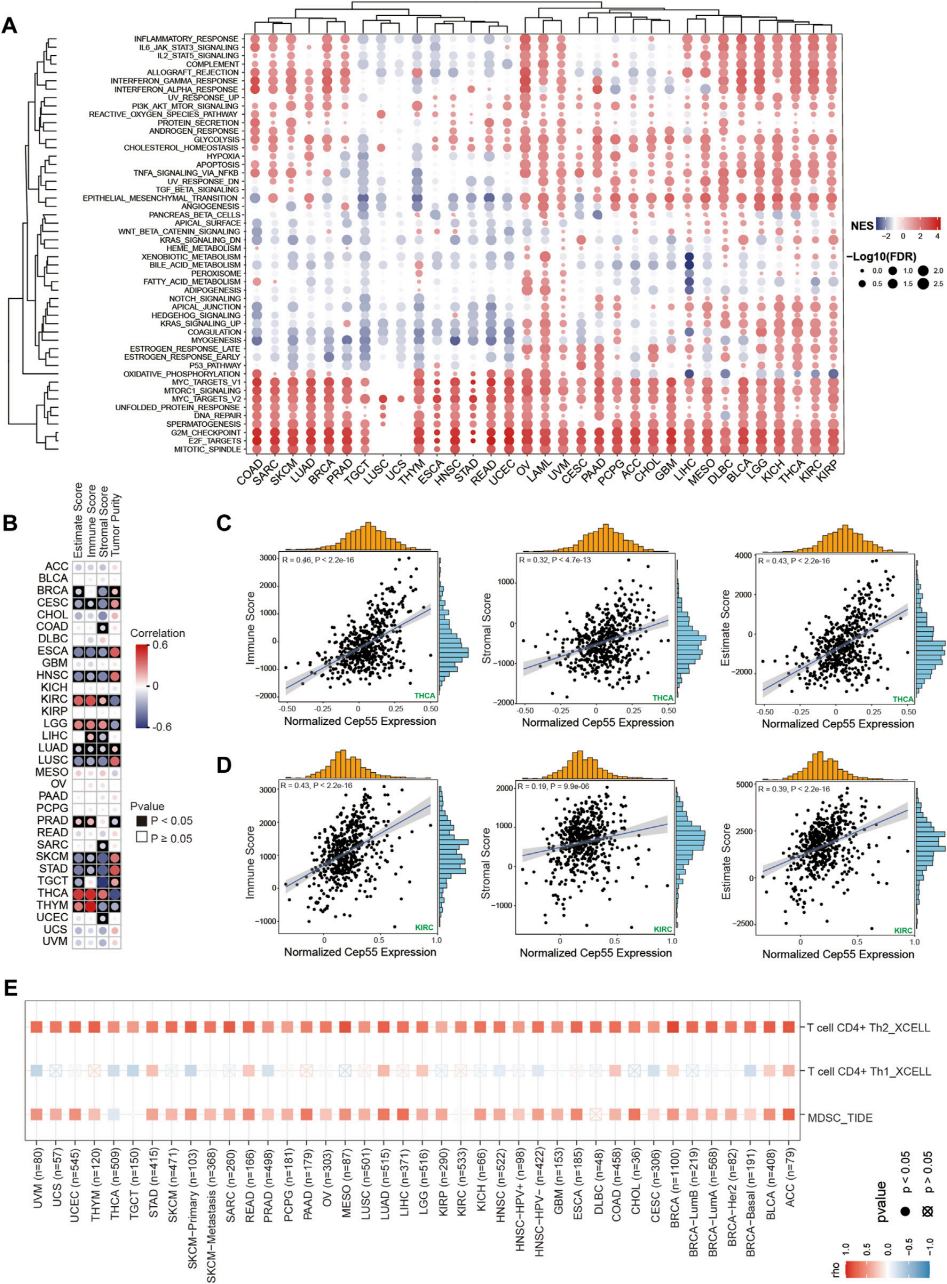
Gene set enrichment analysis (GSEA) and immune infiltration analysis in pan-cancer. **(A)** Heatmap of enrichment scores. The size of the circle represents the value of the false discovery rate (FDR), and the color represents the normalized enrichment score (NES). **(B)** The correlations between CEP55 expression and estimated scores. Red, positive correlation; Blue, negative correlation. **(C–D)** Scatter plots of correlations between CEP55 expression and immune, stromal, and estimated scores in THCA and KIRC. **(E)** Correlations between CEP55 expression and infiltration abundances of Th2 cells, Th1 cells, and MDSCs.

To further elucidate the biological role of CEP55 in cancer, a differential expression analysis was performed between high- and low-CEP55 expression groups for each cancer type. Gene set enrichment analysis (GSEA) was then performed using the different expression genes (DEGs) for each malignancy. Cancers with high CEP55 levels were considerably enriched in cell cycle- and proliferation-related pathways, such as the mitotic spindle, E2F targets, and G2M checkpoints, indicating that the malignancies were in a high proliferation state. Furthermore, we discovered that immune-related pathways, such as the IL6-JAK-STAT3 signaling, inflammatory response, IFNα response, and IFNγ response pathways, were significantly enriched in high-CEP55 malignancies, particularly for KICH, LGG, THCA, and LIHC (Figure 5A; Supplementary Table S5). These findings suggest that CEP55 is involved in tumor invasion and progression, as well as immunological responses.

### 3.6 CEP55 expression correlates with tumor immune cell infiltration

Tumor-infiltrating immune cells are directly involved in the occurrence, progression, and metastasis of malignancies. The GSEA revealed a correlation between CEP55 expression and immune-related pathways. We further explored the relationships between the expression of CEP55 and the abundance of tumor-infiltrating immune cells. First, we calculated the immune and stromal scores using the ESTIMATE algorithm. CEP55 expression was significantly correlated with immune scores in ESCA, CESC, HNSC, LGG, KIRC, LIHC, LUSC, LUAD, PRAD, SKCM, THCA, STAD, and THYM (Figures 5B–D, *p* < 0.05; Supplementary Table S6). Furthermore, to investigate the immune cells that were correlated with CEP55, we used the TIMER2.0 database, which estimates the abundance of diverse immune cells using multiple methods. The pan-cancer immune cell invasion is shown in Supplementary Figure S4. The majority of the malignancies showed substantial correlations between the expression of CEP55 and the presence of myeloid-derived suppressor cells (MDSCs) and T helper 2 (Th2) cells (Figure 5E). CEP55 expression was positively correlated with the number of MDSCs infiltrating the malignancies, including ACC (Cor = 0.769, *p* = 2.07e-15), LIHC (Cor = 0.69, *p* = 5.48e-50), and LUAD (Cor = 0.656, *p* = 5.05e-62). Moreover, CEP55 expression was positively correlated with the infiltration abundance of Th2 cells in ACC (Cor = 0.809, *p* = 4.86e-18), BLCA (Cor = 0.778, *p* = 9.62e-76), BRCA (Cor = 0.873, *p* = 1.45e-311), BRCA-LumA (Cor = 0.745, *p* = 1.17e-92), BRCA-LumB (Cor = 0.715, *p* = 2.31e-31), LUAD (Cor = 0.762, *p* = 6.73e-95), MESO (Cor = 0.821, *p* = 6.21e-22), READ (Cor = 0.703, *p* = 1.11e-14), SARC (Cor = 0.79, *p* = 2.86e-53), SKCM-Primary (Cor = 0.773, *p* = 1.91e-21), SARC (Cor = 0.764, *p* = 3.26e-23), and UCEC (Cor = 0.716, *p* = 5.60e-15). These profiles show that CEP55 expression is selectively correlated with the infiltration of immune cell populations into tumors and may serve as a key regulator in the tumor microenvironment.

### 3.7 CEP55 correlates with immunomodulators and predicts response to cancer immunotherapy

**FIGURE 6:**
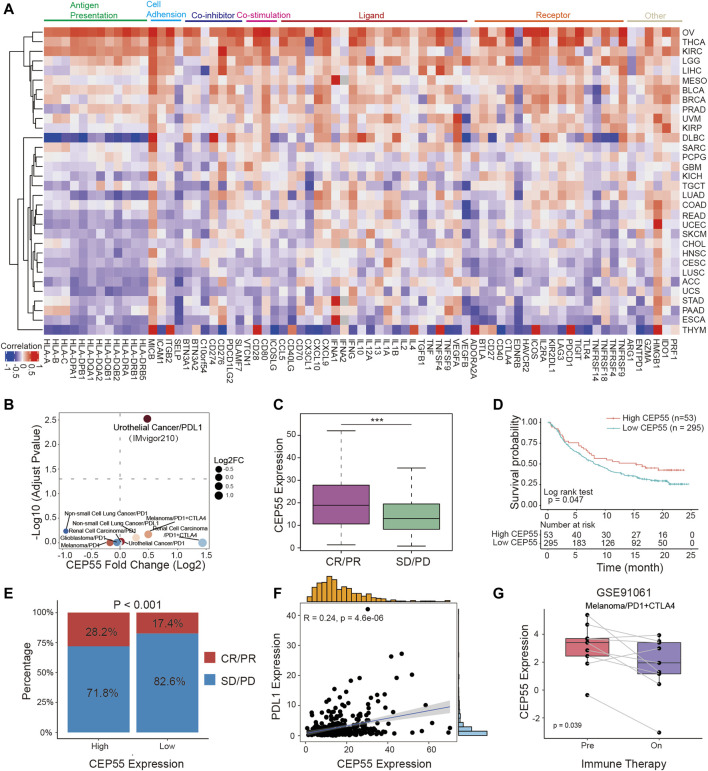
CEP55 in association with immunomodulators predicts cancer immunotherapy response. **(A)** Heatmap of correlation efficiency of CEP55 with immunoregulators. Red, positive correlation; Blue, negative correlation. **(B)** Differences in CEP55 expression between responders and non-responders in multiple immunotherapy cohorts. **(C)** CEP55 expression in CR/PR and SD/PD patients of the IMvigor210 cohort. **(D)** Kaplan-Meier curve of the low and high-CEP55 subgroups in the IMvigor210 cohort. **(E)** The proportion of responders and non-responders in the high and low-CEP55 expression groups of the IMvigor210 cohort. **(F)** The correlation between CEP55 expression and PDL1 expression in the IMvigor210 cohort. **(G)** CEP55 expression changes after ICI treatment in the GSE91061 cohort. Asterisks indicate statistical *p*-values (ns, *p* > 0.05, *p < 0.05, **p < 0.01, and ***p < 0.001).

To better characterize the mechanistic relationship between CEP55 and the tumor immunological milieu, we assessed the correlation between CEP55 expression and a collection of immunomodulators (Thorsson et al., 2018). CEP55 expression was positively correlated with most immunomodulatory variables in OV, THCA, KIRC, LGG, LIHC, MESO, BLCA, BRCA, PRAD, and UVM, but negatively correlated with STAD, PAAD, ESCA, and THYM (Figure 6A; Supplementary Table S7). We further studied whether CEP55 expression affected patient response to ICIs by detecting the differences in CEP55 expression between ICI response (CR/PR) and non-response (SD/PD) patients. For urinary tumors treated with PDL1 inhibitors (the IMvigor210 cohort), CEP55 expression differed significantly between the CR/PR and SD/PD groups (Figure 6B), with the CR/PR group exhibiting higher CEP55 expression than the SD/PD group (Figure 6C). When patients with urinary malignancies were treated with a PDL1 inhibitor, those with high CEP55 expression outlived the patients with low CEP55 expression (Figure 6D). Patients with high CEP55 expression responded to the PDL1 inhibitor at a rate of 28.2%, which was considerably greater than the 17.4% shown in patients with low CEP55 expression (Figure 6E). CEP55 and PDL1 expression showed a strong positive correlation (Figure 6F). Moreover, in the GSE91061 melanoma cohort treated with PD1 and CTLA4 inhibitors, CEP55 expression decreased considerably after ICI treatment in the CR/PR group (Figure 6G). These findings indicate that, in addition to its role in carcinogenesis, as previously documented, CEP55 may also be involved in the modulation of the tumor immune microenvironment; therefore, it has the potential as a biomarker to predict immunotherapy response.

### 3.8 Construction and validation of CEP55-Related risk model in HCC

**FIGURE 7:**
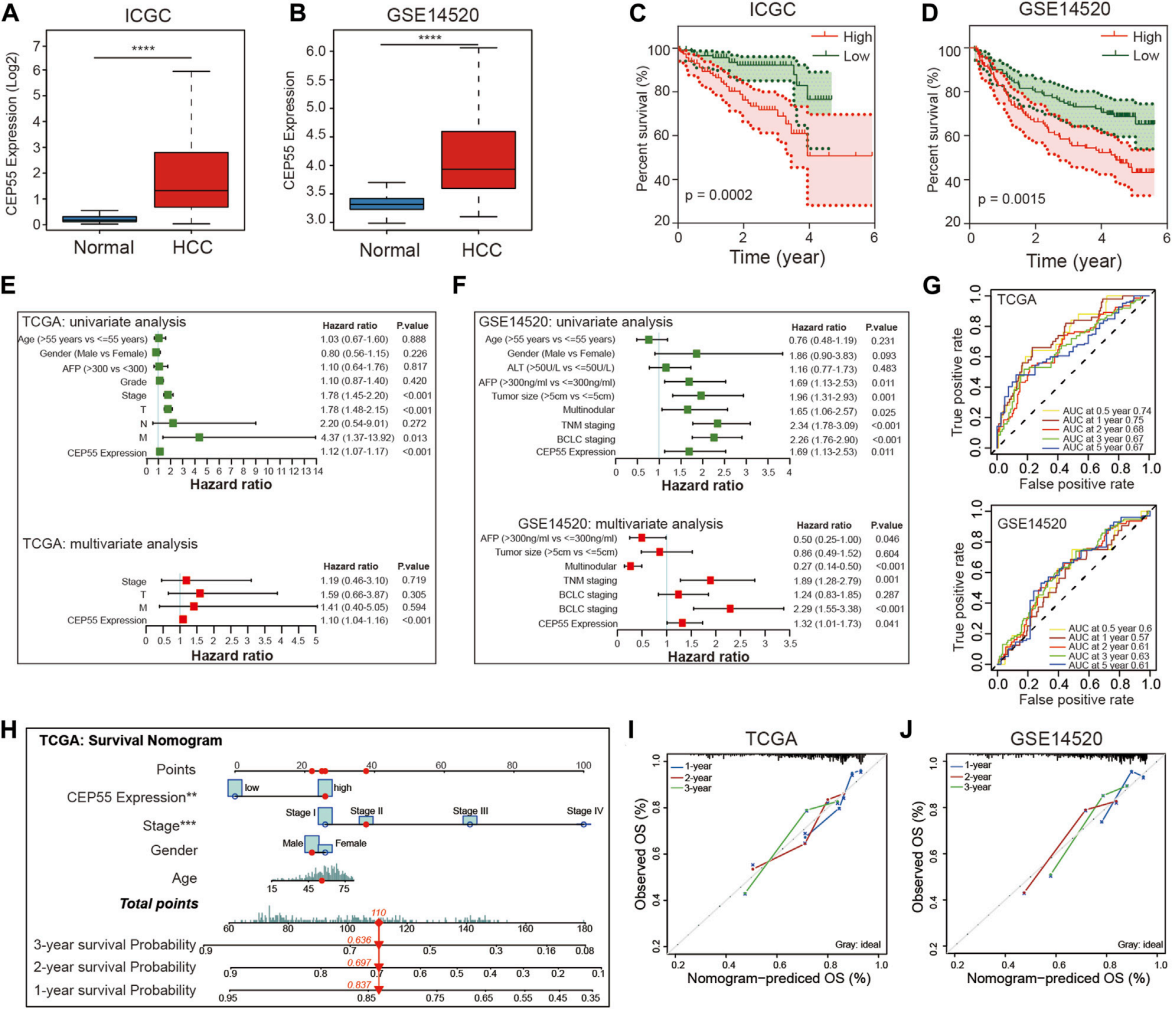
Validation of CEP55 expression and construction of prognostic risk model for HCC. **(A-B)** CEP55 expression in normal and tumor tissues in the ICGC and GSE14520 datasets. **(C-D)** Kaplan-Meier curve of the high and low CEP55 expression groups in the ICGC and GSE14520 datasets. **(E-F)** Univariate and multivariate Cox regression analyses for CEP55 expression and clinical features in TCGA and GSE14520 datasets. **(G)** Time-dependent ROC curve analysis to assess the predictive efficacy of CEP55 in TCGA and GSE14520 datasets. **(H)** Nomogram for quantitatively predicting the probability of 1-, 2-, and 3-year OS for HCC patients. **(I-J)** Calibration plots of nomogram in TCGA and GSE14520 datasets. Asterisks indicate statistical *p*-values (ns, *p* > 0.05, *p < 0.05, **p < 0.01, and ***p < 0.001).

Two recent studies have identified certain functions of CEP55 in HCC. Li et al. demonstrated that CEP55 regulated the JAK2-STAT3-MMPs signaling pathway and promoted HCC cell migration and invasion (Li et al., 2018). Yang et al. revealed that CEP55 participated in SPAG5-mediated proliferation and migration of HCC cells and provided a viable therapeutic target for the clinical treatment of HCC (Yang et al., 2018). These findings indicate that increased CEP55 expression could be particularly important in the context of HCC. Therefore, we investigated the effects of CEP55 on HCC. Consistent with previous findings, CEP55 was documented to be more abundant in tumor tissues than in non-tumor tissues in both the ICGC and GSE14520 datasets (Figures 7A,B). In addition, the group with high CEP55 expression had a lower survival rate (Figures 7C,D). The prognostic effect of CEP55 in HCC was further examined using univariate and multivariate Cox regression analyses of HCC datasets. The univariate (HR = 1.12, *p* < 0.001) and multivariate (HR = 1.10, *p* < 0.001) analyses of TCGA LIHC dataset showed that CEP55 expression was significantly correlated with prognosis. Similar outcomes were found using the GSE14520 dataset with univariate (HR = 1.69, *p* = 0.011) and multivariate (HR = 1.32, *p* = 0.041) Cox regression analyses (Figures 7E,F). Moreover, a time-dependent ROC curve analysis was performed to evaluate the predictive classification efficiency of CEP55 expression in HCC. In TCGA dataset, the area under the curve (AUC) values for 0.5-, 1-, 2-, 3-, and 5-year overall survival were 0.74, 0.75, 0.68, 0.67, and 0.67, respectively, whereas the corresponding values of the GSE14520 dataset were 0.6, 0.57, 0.61, 0.63, and 0.61 (Figure 7G). These results indicate that CEP55 expression may help predict the short- and long-term survival status of patients with HCC. We then merged the separate prognostic markers to create a nomogram that doctors can use as a quantitative technique to predict death in HCC patients (Figure 7H). Each patient can be assigned a total score by adding the scores for each prognostic criterion. Higher overall scores would indicate a worse prognosis for the patient. In addition, the calibration graphs of several cohorts (TCGA and GSE14520) revealed that the nomogram performed similarly to the ideal model and was capable of explaining a large portion of patient outcomes (Figures 7I,J).

### 3.9 CEP55 expression is linked to HCC subclass

**FIGURE 8:**
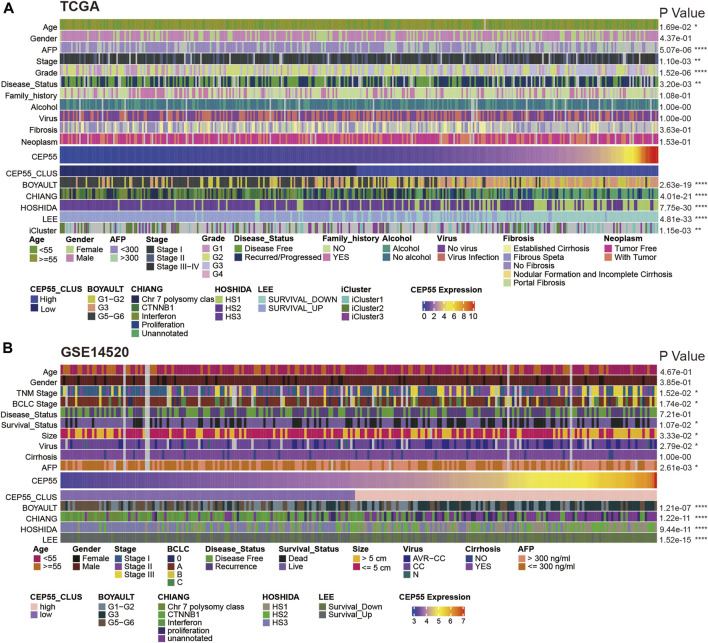
Relationships between CEP55 expression and HCC molecular subtypes in **(A)** TCGA and **(B)** GSE14520 datasets. Asterisks indicate statistical *p*-values (ns, *p* > 0.05, *p < 0.05, **p < 0.01, and ***p < 0.001).

Subsequently, using data from TCGA LIHC cohort and the GSE14520 dataset, we examined the association of tumor-associated clinicopathological characteristics with CEP55 expression (Figures 8A,B). These components were significantly correlated in HCC, based on the chi-squared test. In TCGA cohort, CEP55 expression was correlated with age, serum AFP level, pathologic stage, histologic grade, and tumor recurrence (*p* < 0.05). Similarly, in the GEO cohort, CEP55 expression was associated with tumor stage, survival status, tumor size, viral infection, and serum AFP level (*p* < 0.05). Several studies have reported that molecular-based stratification of HCC could identify disease subtypes with differential outcomes (Lee et al., 2004; Boyault et al., 2007; Hoshida et al., 2009), and the relationships between CEP55 expression and these subtypes were analyzed. In TCGA cohort, increased CEP55 expression was substantially related to Boyault’s G3 subclass (*p* < 0.001), Chiang’s unannotated subclass (*p* < 0.001), Hoshida’s1 subclass (*p* < 0.001), Lee’s Survival_Down subclass (*p* < 0.001), and TCGA iCluster1 subclass (*p* < 0.001). Low CEP55 expression was associated with Boyault’s G5/G6 subclass (*p* < 0.001), Chiang’s CTNNB1 subclass (*p* < 0.001), Hoshida’s2 subclass (*p* < 0.001), Lee’s Survival_Up subclass (*p* < 0.001), and TCGA iCluster2 subclass (*p* < 0.001). Similarly, increased CEP55 expression in the GEO cohort was substantially related to Boyault’s G3 subclass (*p* < 0.001), Chiang’s unannotated subclass (*p* < 0.001), Hoshida’s1 subclass (*p* < 0.001), and Lee’s Survival_Down subclass (*p* < 0.001). Low CEP55 expression was associated with Boyault’s G5/G6 subclass (*p* < 0.001), Chiang’s CTNNB1 subclass (*p* < 0.001), Hoshida’s2 subclass (*p* < 0.001), and Lee’s Survival_Up subclass (*p* < 0.001). Based on these results, we showed that the expression of CEP55 may predict an HCC prognosis and that CEP55 exhibits diverse patterns of expression in various molecular subtypes of this disease.

### 3.10 Prediction and validation of potential therapeutic compounds

**FIGURE 9:**
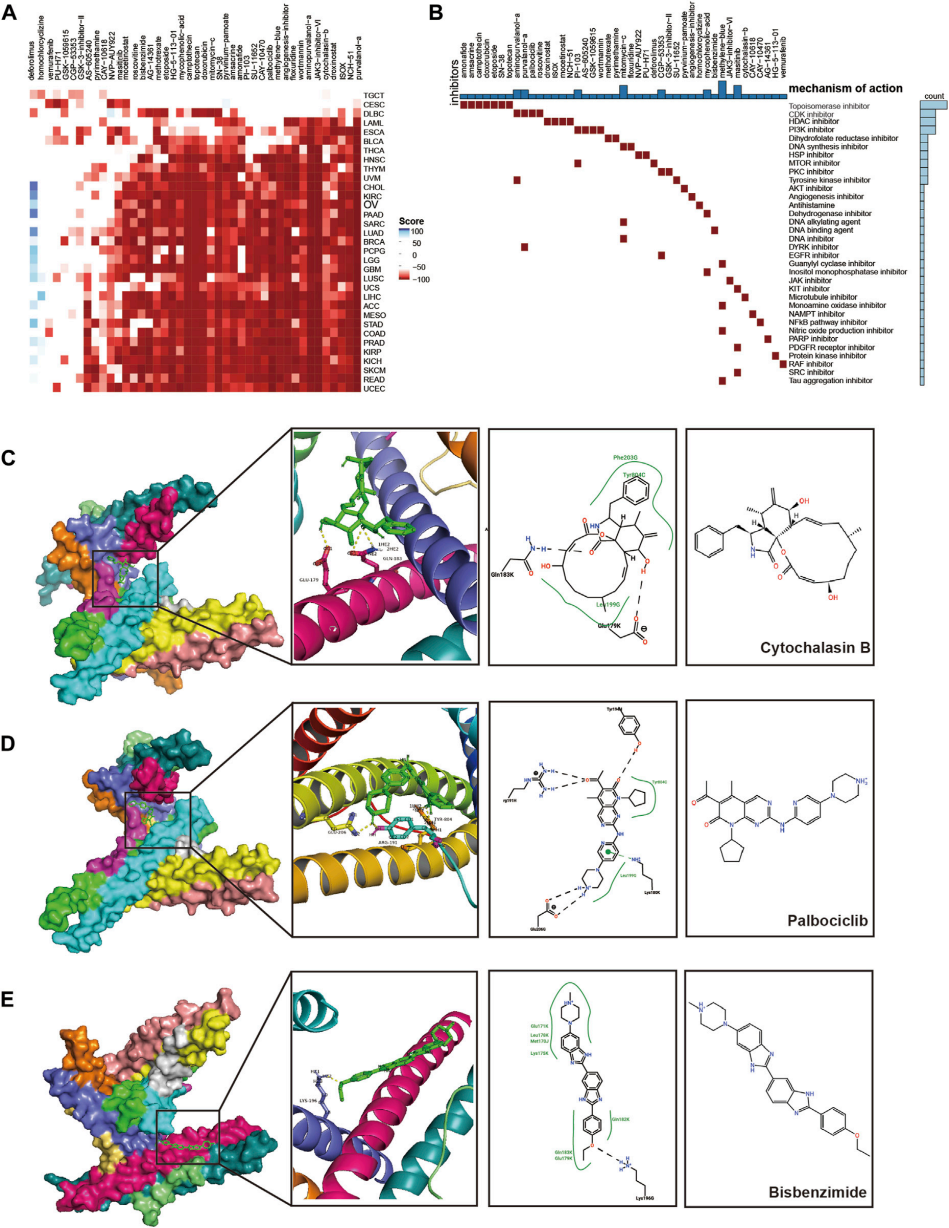
Prediction and validation of potential therapeutic compounds. **(A)** Heatmap of scores for each drug in the CMAP database for various cancers. Red, negative score; Blue, positive score. **(B)** The mechanisms of action (MoA) that were shared by the compounds. **(C–E)** Docking position of the CEP55 predicted active pocket with cytochalasin B, palbociclib, and bisbenzimide.

We screened CEP55-associated inhibitors and components for each cancer type using the connectivity map (CMap) database. Various substances, including purvalanol-A, NCH-51, and ISOX, demonstrated therapeutic potential against CEP55 expression (Figure 9A; Supplementary Table S8). Topoisomerase, CDK, HDAC, and PI3K inhibitors were common mechanisms among small-molecule drugs (Figure 9B). We used a molecular docking approach to determine whether these small molecules could attach to CEP55. Cytochalasin B and CEP55 had a binding free energy of −7.16, and cytochalasin B formed hydrogen bonds with CEP55 via GLU-179 and GLN-183 (Figure 9C). Palbociclib and CEP55 had a binding free energy of −7.38 and hydrogen bonds were formed between these through GLU-206, ARG-191, and TRY-804 (Figure 9D). Bisbenzimide and CEP55 showed a binding free energy of −7.00, and these hydrogen bonds were created via LYS-196 (Figure 9E). Thus, these clinically available compounds could bind CEP55, and further studies should be conducted to evaluate the outcomes of CEP55 inhibition in a tumor context. The design of new molecular inhibitors, particularly proteolysis-targeting chimeras, may also be able to use these as reference scaffolds.

## 4 Discussion

CEP55 has been identified as a critical factor in cellular abscission, and further research revealed that it is abnormally expressed in various cancers and is implicated in cancer cell proliferation, invasion, and migration (Jeffery et al., 2016). CEP55 knockdown arrests the cell cycle at the G2/M phase and suppresses gastric cancer cell expansion (Tao et al., 2014). Furthermore, CEP55 drives the migration and invasion of oral cavity squamous cell carcinoma by increasing FOXM1 and MMP-2 activity (Chen et al., 2009). Recently, researchers demonstrated that CEP55 promoted the epithelial-mesenchymal transition (EMT) and activated the PI3K/AKT/mTOR pathway in renal cell cancer (Chen et al., 2019). Collectively, these reports show that apart from regulating the cell cycle, CEP55 may also actively participate in the central cell processes required for the occurrence of malignancies. The possible involvement of CEP55 in the tumor microenvironment warrants further investigation.

In the present study, we investigated CEP55 expression and its relationship with pan-cancer prognoses. CEP55 expression was revealed to be considerably higher in tumor tissues than non-cancerous tissues in the following cancers: ACC, BRCA, BLCA, COAD, CESC, CHOL, DLBC, GBM, ESCA, KICH, KIRP, KIRC, LIHC, LUSC, LUAD, OV, READ, PAAD, SKCM, SARC, STAD, THCA, and UCEC. In addition, overexpression of this protein has been reported in association with numerous malignancies, including gastric carcinoma, lung cancer, renal cell cancer, breast cancer, and esophageal squamous cell carcinoma (Ma et al., 2003; Jones et al., 2005; Sakai et al., 2006; Yan et al., 2021). CEP55 is involved in multiple malignancy risk signatures for predicting cancer prognosis, progression, and chemotherapy response, and is within the main 70 overexpressed genes in cancers with chromosomal instability (the CIN70 signature) that are frequently used to predict clinical prognoses and distant metastases (Carter et al., 2006). CEP55 is also part of the PAM50 signature, which is used for breast cancer categorization and prognostic prediction. Our findings show that CEP55 may be a carcinogenic indicator and a viable prognostic biomarker for a variety of malignancies.

In addition to its involvement in cell cycle and proliferation-related pathways, this study showed that CEP55 was closely associated with immune-related pathways, including the IL6-JAK-STAT3 signaling and the IFNα/γ response pathways. In addition, this protein is highly expressed in the lymph nodes, tonsils, and other immune-associated tissues. This suggests that CEP55 could have a more active role in immune modulation than those previously reported. We discovered that abnormal CEP55 expression was substantially linked to the invasion of MDSCs and Th2 cells in most malignancies. MDSCs have a diverse population of cells that are descended from myeloid cells, and activated MDSCs produce the immune-suppressive factors arginase 1, iNOS, and ROS (Gabrilovich and Nagaraj, 2009). T-helper 1 (Th1) and Th2 cells are the major functional subsets of CD4^+^ T cells (Chatzileontiadou et al., 2020; Zhu and Zhu, 2020), and the two subgroups differ in terms of cytokine production and immunological responses. Th1 cells boost the cytotoxic effects of NK and CD8^+^ T cells by secreting Th1-type cytokines such as TNFα, IL2, and IFNγ (Mosmann and Coffman, 1989; Murphy et al., 2000; Alspach et al., 2019), whereas Th2 cells produce immunosuppressive cytokines, including IL-4, IL-5, IL-10, and IL-13. Th2-type cytokines inhibit Th1 cell development and the immune response, thereby limiting antitumor immunity (Protti and De Monte, 2012; Nakayama et al., 2017; Basu et al., 2021). Balancing Th1/Th2 cells is essential for maintaining immune homeostasis, and a change in this ratio toward increased Th2 cell infiltration could promote cancer development and weaken the immune system (Kidd, 2003; Ruterbusch et al., 2020). According to the results of this investigation, CEP55 expression was positively linked to Th2 cell infiltration, suggesting that CEP55 may be the catalyst or promoter of this Th1/Th2 balance shift. In addition, the current study indicated that CEP55 could be implicated in the modulation of the immunosuppressive microenvironment in malignancies and can be a marker for predicting responses to ICIs. However, the fundamental mechanisms by which CEP55 participates in immune modulation and immunotherapy responses remain unknown.

HCC is the fourth leading cause of cancer-related mortality globally and the most common primary liver cancer (Villanueva, 2019). The elevated CEP55 expression in tumor tissue and its association with poor survival in HCC identified in this study corroborate previously published results. Overexpression of this protein causes AKT phosphorylation and activation, which promotes survival and tumor formation in HCC (Chen et al., 2007). HCC is a heterogeneous malignancy of hepatocytes that is characterized by the accumulation of many genomic and epigenomic changes that have undergone Darwinian selection. Effective precision medicine for HCC therapy can be achieved by the accurate molecular subtyping of HCC (Rebouissou and Nault, 2020). We demonstrated that distinct hepatocellular carcinoma molecular subtypes can be identified based on CEP55 expression. Owing to the high levels of CEP55 expression in exosomes, CEP55 may be a viable biomarker for liquid biopsies to predict survival and direct precision medicine for HCC patients. Moreover, CEP55 expression was significantly inversely associated with specific metabolic pathways in HCC, such as xenobiotic and bile/fatty acid metabolism. Metabolic reprogramming has been identified as one of the variables driving cancer aggression and affecting neoadjuvant chemotherapy response (Wu et al., 2022). Therefore, a greater understanding of the function of CEP55 in metabolic regulation is required.

We detected possible inhibitors for the regulation of CEP55 expression or the targeting of CEP55-related pathways and confirmed these using molecular docking analyses. Three small-molecule compounds were identified that had a strong affinity for CEP55: cytochalasin B, which is a G-actin superimposed inhibitor that can suppress the growth of many types of cancer (Croop and Holtzer, 1975); palbociclib, which is a CDK4/6 inhibitor licensed by the FDA for the treatment of ER+, HER2-breast cancer (Konar et al., 2022), and given that CDK4/6 are critical cell cycle regulators, off-target effects of palbociclib on CEP55 may contribute to its efficacy in triggering tumor cell cycle arrest; and bisbenzimide, which is involved in numerous pharmacological actions, including anticancer, antiparasitic, antibacterial, antifungal, antiviral, and chemosensor activities (Verma et al., 2020). However, the exact effects of these three small compounds on CEP55 remain to be further investigated. We expect that by binding to CEP55, these three molecules will alleviate the immunosuppressive microenvironment and enhance the antitumor activity of ICIs.

Several studies have investigated the specific roles of CEP55 in malignancies. Fu et al. demonstrated that CEP55 was a diagnostic and predictive factor in patients with LUAD and LUSC (Fu et al., 2020). Yang et al. discovered that CEP55 was overexpressed in liver cancer tumor tissue and linked to poor prognoses and enhanced immune infiltration (Yang et al., 2020). However, these studies did not thoroughly analyze the role of CEP55 in the tumor immune microenvironment. Yang et al. found that CEP55 was correlated with the infiltration of B cells, CD4^+^ T cells, CD8^+^ T cells, macrophages, neutrophils, and dendritic cells in liver cancer. Through our pan-cancer analysis, we found that CEP55 was significantly correlated with MDSCs and Th1 cells in most cancers and positively correlated with the expression of checkpoints, such as PD1, CTLA4, LAG3, and TIGIT in certain cancer types. This indicates that CEP55 overexpression may be a driving factor for CD8^+^ T cell exhaustion. Furthermore, we used molecular docking analysis to predict and validate chemicals that could bind to CEP55, which has not been previously reported.

This study had some limitations. Although we validated the results of this work using a large number of datasets, additional experiments and clinical investigations are required to identify the precise function of CEP55 in carcinogenesis and development. Furthermore, we discovered that the expression of CEP55 is associated with an imbalance in Th1/Th2 cells; however, it is unclear how CEP55 controls the differentiation of Th1 and Th2 cells and additional research is required to elucidate the underlying processes. In addition, the potential of tumor-expressing CEP55 as a TAA for modulating immune activity should be explored, possibly through the identification and clonal tracing of CEP55 antigen-specific T cells in the context of human tumors.

In conclusion, our analysis provides a comprehensive assessment of CEP55 expression in various cancers. CEP55 is a powerful tumor prognostic marker that is involved in tumor immune modulation. Therefore, CEP55 has potential as a therapeutic target.

## Data Availability

The original contributions presented in the study are included in the article/Supplementary Material, further inquiries can be directed to the corresponding authors.
