# Supplementary figures and images for: Cancer-testis antigen CEP55 serves as a prognostic biomarker and is correlated with immune infiltration and immunotherapy efficacy in pan-cancer
Source: Front Mol Biosci. 2023 Jul 7;10:1198557. doi: 10.3389/fmolb.2023.1198557 (PMC10360201; doi:10.3389/fmolb.2023.1198557)

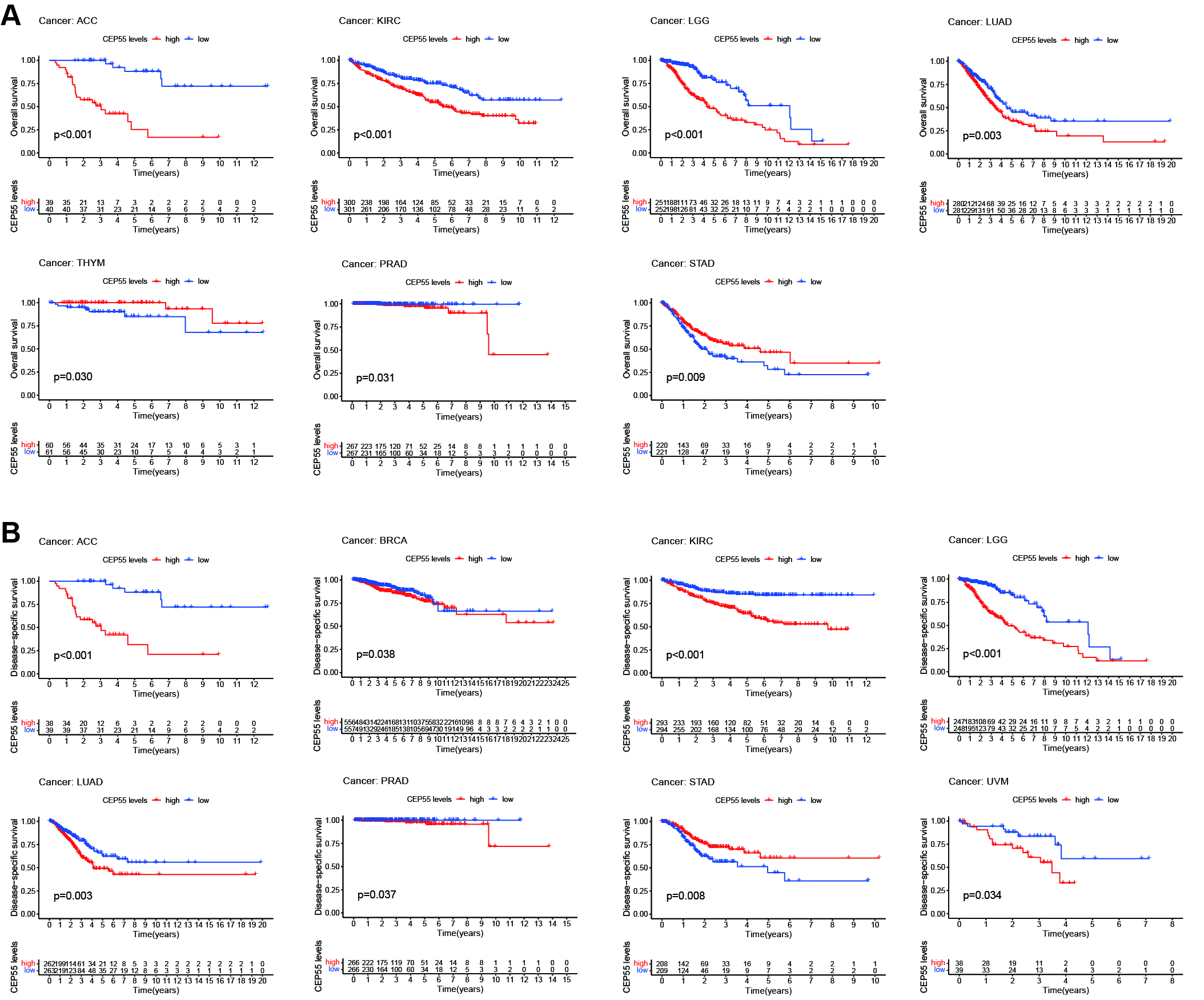

Supplement: Supplementary file 1 [file Image3.TIF]

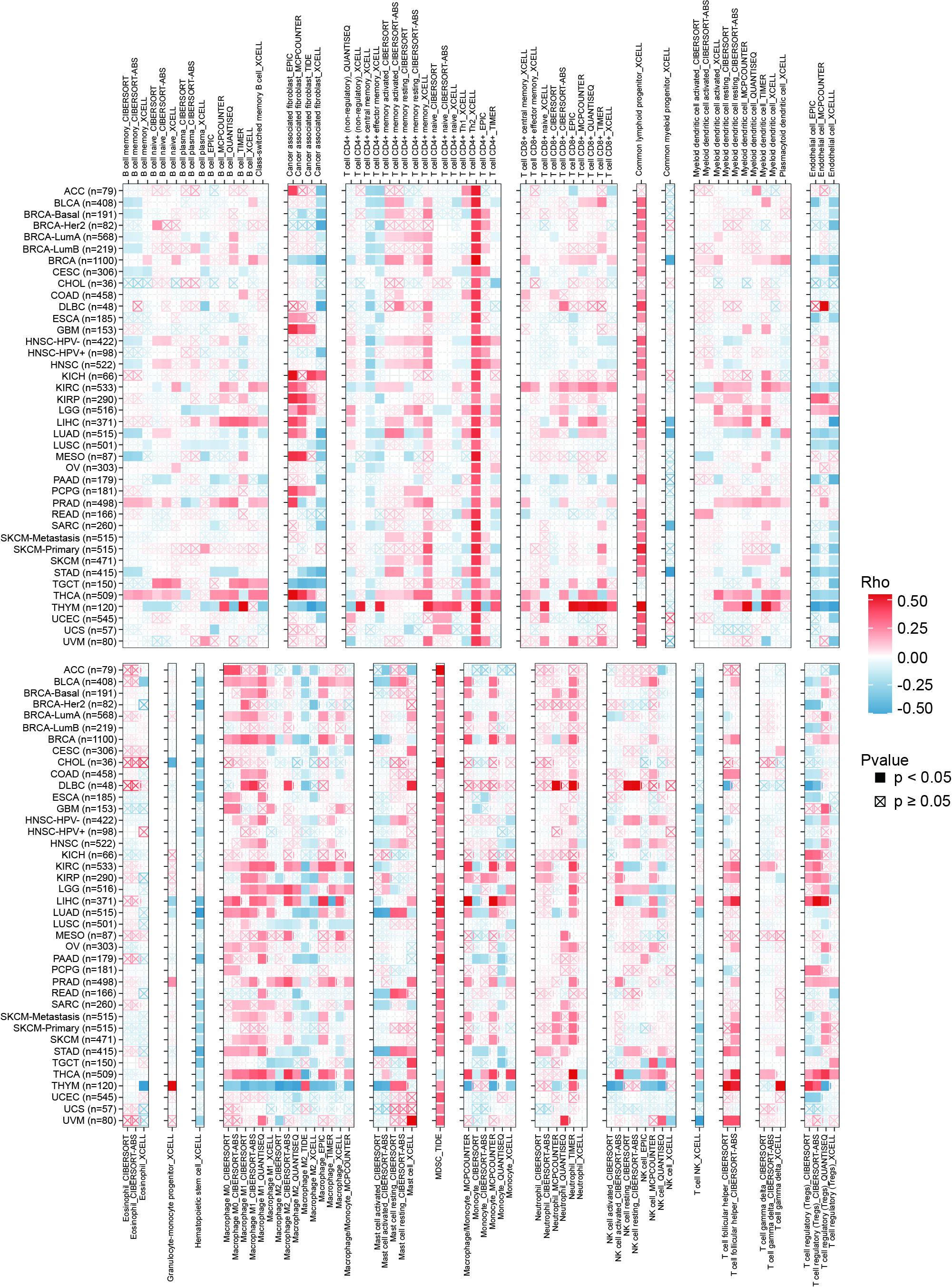

Supplement: Supplementary file 2 [file Image4.TIF]

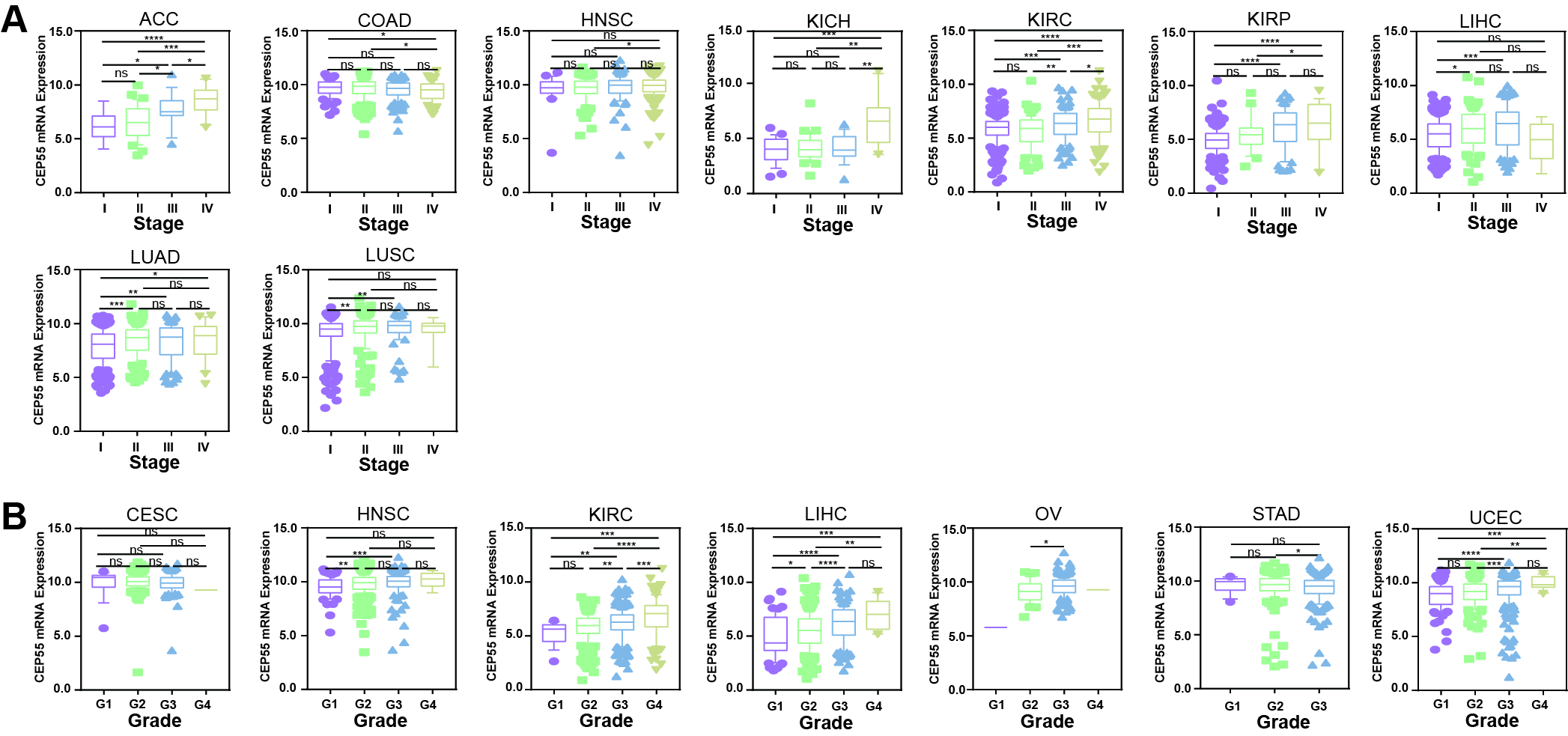

Supplement: Supplementary file 3 [file Image2.TIF]

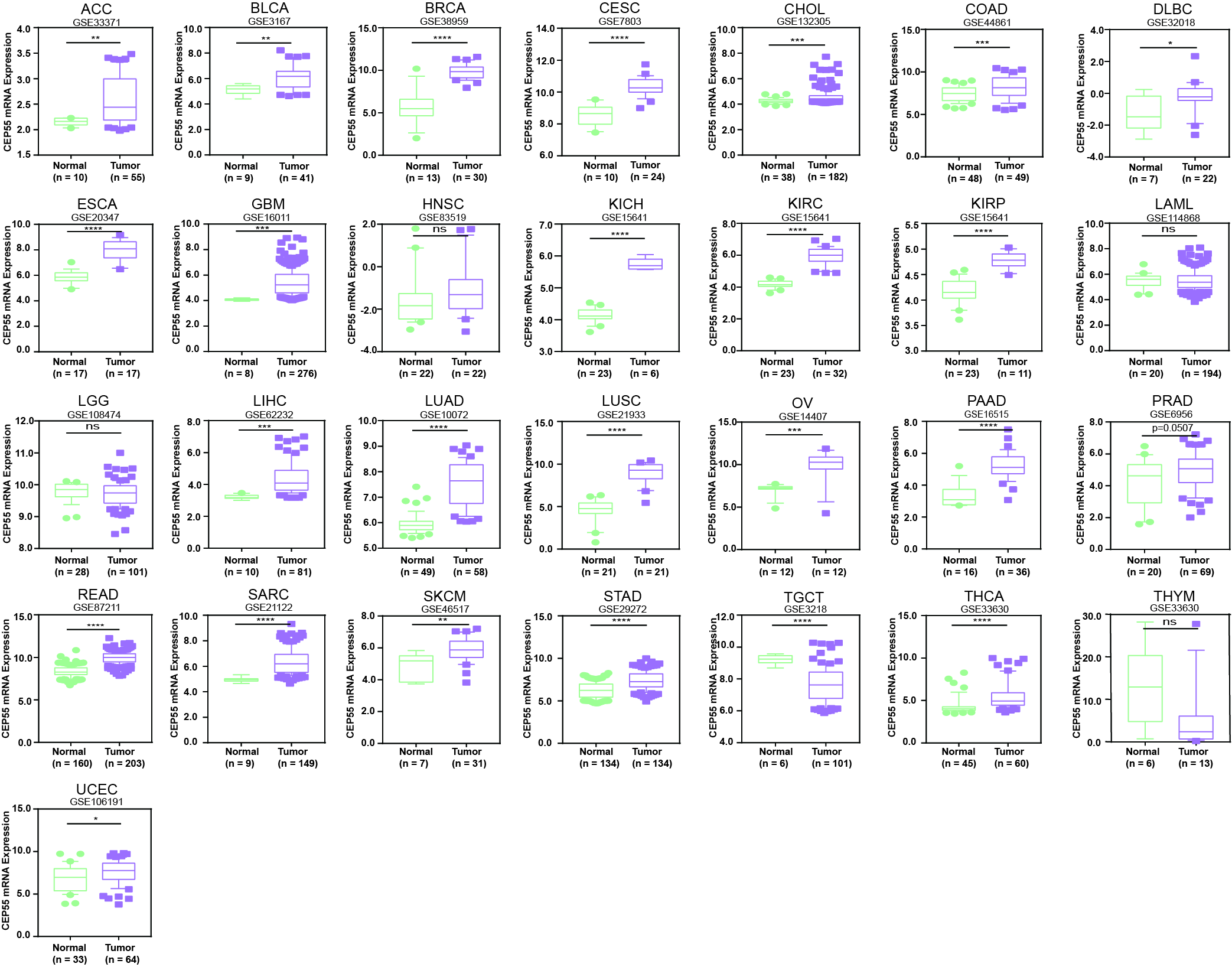

Supplement: Supplementary file 4 [file Image1.TIF]
